# The role of repeat fine needle aspiration in the management of indeterminate thyroid nodules

**DOI:** 10.1186/s40463-016-0164-0

**Published:** 2016-10-18

**Authors:** Alborz Jooya, Joe Saliba, Audrey Blackburn, Michael Tamilia, Michael P. Hier, Alex Mlynarek, Véronique-Isabelle Forest, Louise Rochon, Anca Florea, Hangjun Wang, Richard J. Payne

**Affiliations:** 1 Department of Otolaryngology - Head and Neck Surgery – Jewish General Hospital, McGill University, 3755 Chemin de la Côte-Sainte-Catherine, Room E-903, Montréal, H3T 1E2 QC Canada; 2 Division of Endocrinology, Jewish General Hospital - McGill University, Montreal, QC Canada; 3 Department of Pathology, Jewish General Hospital - McGill University, Montreal, QC Canada

**Keywords:** Ultrasound Guided Fine Needle Aspiration, FNA, Bethesda, Repeat FNA

## Abstract

**Background:**

Management decisions are not straightforward when the Ultrasound Guided Fine Needle Aspiration (USFNA) demonstrates a Bethesda score of either category III or IV, and a diagnostic hemi-thyroidectomy or a repeat USFNA (r-USFNA) could be performed. The aim of this study is to assess the effectiveness of r-USFNA in the management of indeterminate thyroid nodules by evaluating the likelihood of obtaining a definite diagnosis.

**Methods:**

We reviewed the medical records of all patients with thyroid nodules between 2011 and 2015 at the Jewish General Hospital (Montreal, Canada). Three hundred fifty-one patients who had undergone a surgical procedure (hemi or total thyroidectomy) and a diagnosis of B3 or B4 on the primary USFNA (p-USFNA) were included in the study. Ninety-six of the included patients also had a repeat USFNA prior to the surgery. Demographic data, type of procedure, and McGill Thyroid Nodule Score (MTNS) were obtained from the medical records. Malignancy rates were calculated based on the final surgical histopathology report.

**Results:**

Upon r-USFNA, an average 76 % of patients did not change Bethesda categories, 7.4 % downgraded to a benign category. The results showed that, on an average 17.3 % of patients with p-USFNA of B3 and 20 % of patients with p-USFNA of B4, upgraded to a malignant or suspicious for malignancy category, thus changing the clinical management to total thyroidectomy. Our data demonstrates that r-USFNA facilitates choosing the correct surgery of total thyroidectomy in about 20 % of nodules that have upgraded from B3/B4 to a more definite malignant category.

**Conclusions:**

r-USFNA in patients with indeterminate diagnoses (B3 or B4) increases categorization into more definite categories. Approximately 20 % of patients are found to have malignant thyroid nodules and suspicious for malignancy thyroid nodules upon repeating the biopsy, hence a diagnostic hemi-thyroidectomy was avoided and a more definitive surgery could be performed. Furthermore, repeat USFNA results in a fewer number of hemi-thyroidectomy and completion thyroidectomy procedures.

## Background

Ultrasound Guided Fine Needle Aspiration (USFNA) is a cost-effective and highly reliable procedure used to evaluate and guide the management of thyroid nodules [1]. The Bethesda System for Reporting Thyroid Cytopathology (BSRTC) has resulted in standardization, reproducibility and improved clinical significance of thyroid USFNA. It is comprised of the following six categories: B1, non-diagnostic; B2, benign; B3, Atypia of undetermined significance or follicular lesion of undetermined significance; B4, (suspicious for) Follicular neoplasm; B5, Suspicious for malignancy; B6, malignant. Management decisions are not straightforward when the USFNA demonstrates a Bethesda score of either category III (B3) or IV (B4) [2–6]. In those instances, it is not uncommon for some patients to undergo diagnostic hemi-thyroidectomy to obtain a definitive diagnosis, while others prefer a more conservative approach and opt for a repeat USFNA (r-USFNA). However, none of these scenarios are ideal. A hemi-thyroidectomy is rarely the procedure of choice for a thyroid nodule: it is either unnecessary (in the event of a benign lesion) or inadequate (in the event of a malignant lesion). Likewise, the benefit of repeat USFNA is unclear with respect to its contribution to patient management. If the r-USFNA results in Bethesda II (B2), Bethesda V (B5) or Bethesda VI (B6), then management recommendations are more clear and the test is deemed to be useful. However, if the test yields a Bethesda I (B1), B3, or B4, then r-USFNA is not helpful. The aim of this study is to assess the effectiveness of repeat USFNA in the management of indeterminate thyroid nodules (B3 and B4) by evaluating the likelihood of obtaining a definite diagnosis. Moreover, this investigation assesses the usefulness of r-USFNA in the prevention of completion hemi-thyroidectomies in patients with initial B3 or B4 diagnoses.

## Methods

A retrospective chart review of all patients with thyroid nodules classified by BSRTC who underwent a hemi- or total thyroidectomy between 2011 and 2015 at the Jewish General Hospital (Montreal, Canada) was performed. Three hundred fifty-one patients who had undergone a surgical procedure (hemi or total thyroidectomy) and a diagnosis of B3 or B4 on the primary USFNA (p-USFNA) were included in the study. Ninety-six of the included patients also had a repeat USFNA prior to the surgery. Demographic data, type of procedure, and McGill Thyroid Nodule Score (MTNS) were obtained from the medical records. Patients with missing medical data or a diagnosis other than B3 or B4 on p-USFNA were excluded from the study. Malignancy rates were calculated based on the final surgical histopathology report. Fine needle aspirations were performed by otolaryngologists or interventional radiologists under ultrasound guidance using 25- or 27-gauge needle with typically one to four passes.

The decision to undergo thyroidectomy in the patient population was influenced by a high McGill Thyroid Nodule Score (MTNS). MTNS was previously shown to accurately represent the risk of malignancy in a thyroid nodule, given it takes into account a variety of demographic and clinical risk factors in addition to the cytological features evident on USFNA [7]. Other significant operative criteria for our patients included worrisome features on ultrasound, clinical suspicion and patient preference [8]. In addition, diagnostic surgery was recommended to patients with two consecutive diagnoses of B3 or B4 on p-USFNA and r-USFNA.

All statistical analyses were performed using SPSS (IBM Corp., Armonk, NY). This study was approved by the institutional Ethics Review Board (protocol CR15-20).

## Results

A total of 96 patients were retained in this study (Table 1). The mean age at diagnosis was 50.7 years (82.3 % female). Table 1 summarizes the pathological characteristics of the excised nodules. The lesions were categorized according to the final histopathological report as benign (40.6 %) or malignant (59.4 %). Papillary thyroid carcinoma comprised the most common type of histology (58.3 % of all lesions). 56.6 % of nodules were multifocal in histology. In addition, the highest proportion (36.8 %) of malignant thyroid nodules in this study were between 1 and 1.9 cm.

**Table 1:** Patient demographics and pathological characteristics of the excised nodules

Variable	Participants (*n* = 96)
Age, years	
Mean (SD)	50.7 (12.2)
Range	19-75
Gender, %	
Male	17.7
Female	82.3
Nature of lesions, n (%)	
Benign	39 (40.6)
Malignant	57 (59.4)
Type of Lesion, n (%)	
Papillary Carcinoma	56 (58.3)
Follicular Adenoma	25 (26.0)
Other	15 (15.6)
Histology, n (%)	
Unifocal lesion	63 (65.6)
Multifocal lesion	33 (34.4)
Size of Malignant Lesion, n (%)	
size < 1 cm	17 (29.8)
1 = < size < 2 cm	21 (36.8)
2 = < size < 4 cm	17 (29.8)
size > = 4 cm	2 (3.5)

Nodules were reclassified after r-USFNA as suspicious for malignancy or malignant (B5 or B6) in 17.3 % of B3 and 20.0 % of B4 nodules. Moreover, 7.4 % of patients with a score of B3 on p-USFNA had a result of benign lesion (B2) on r-USFNA. The proportion of malignancy on final histopathological reports of lesions categorized as B3 or B4 on primary and repeat USFNAs is shown in Table 2. After p-USFNA, 58.0 % of nodules categorized as B3 and 66.7 % of B4 lesions were found to be malignant. Similarly, 54.6 % of lesions classified as B3 after r-USFNA and 55.7 % of B4 were confirmed to be malignant.

**Table 2:** Proportion of malignancy as a function of Bethesda score for p-USFNA and r-USFNA

Diagnostic study	Bethesda score	Number of cases (%)	Malignant final pathology, n (%)
p-USFNA	B3	81 (84.4)	47 (58.0)
	B4	15 (15.6)	10 (66.7)
r-USFNA	B3	55 (73.3)	30 (54.6)
	B4	18 (26.7)	10 (55.7)

*Abbreviations*: *p-USFNA* primary fine-needle aspiration, *r-USFNA* Repeat fine-needle aspiration, *B3* Bethesda score III, *B4* Bethesda score IV

The distribution of Bethesda categories after r-USFNA for lesions initially diagnosed as either B3 or B4 is demonstrated in Table 3, and the corresponding percentage of malignancy of each category is summarized in Table 4.

**Table 3:** Distribution of repeat USFNA cytology according to initial Bethesda classification

p-USFNA	r-USFNA					
	B2	B3	B4	B5	B6	Total
B3						
Frequency	6	50	11	14	0	81
%	7.4	77.2	13.6	17.3	0	
B4						
Frequency	0	5	7	1	2	15
%	0	33.3	46.7	6.7	13.3	
Total						96

*Abbreviations*: *p-USFNA* primary fine-needle aspiration, *r-USFNA* Repeat fine-needle aspiration, *B2* Bethesda score II, *B3* Bethesda score III, *B4* Bethesda score IV, *B5* Bethesda score V, *B6* Bethesda score VI

**Table 4:** Percent malignancy of individual Bethesda scores after repeat USFNA, as compared with initial USFNA

p-USFNA	r-USFNA	Number of cases (%)	Malignant final pathology, n (%)
B3	B2	6 (7.4)	0 (0.0)
(*n* = 81)	B3/B4	61 (75.3)	33 (54.01)
	B5/B6	14 (17.3)	14 (100.0)
B4	B2	0 (0)	0 (NA)
(*n* = 15)	B3/B4	12 (80)	7 (58.33)
	B5/B6	3 (20)	3 (100.0)

*Abbreviations*: *p-USFNA* primary fine-needle aspiration, *r-USFNA* Repeat fine-needle aspiration, *B2* Bethesda score II, *B3* Bethesda score III, *B4* Bethesda score IV, *B5* Bethesda score V, *B6* Bethesda score VI

In addition, differences in the rates of hemi- and completion thyroidectomies between patients with and without a repeat USFNA has been investigated (Table 5). 255 patients had only one USFNA study and did not undergo a repeat procedure; 121 of these patients had a hemi-thyroidectomy operation (47.45 %). Out of the 96 patients who had a repeat USFNA after the diagnosis of B3 or B4 on p-USFNA, 36 underwent hemi-thyroidectomy (37.50 %). Furthermore, 16 out of 255 patients (6.27 %) without r-USFNA underwent completion thyroidectomy, compared to 3 out of 96 (3.12 %) in patients who obtained a r-USFNA.

**Table 5:** Frequency and percentage of hemi- and completion thyroidectomy operations in patients with one or two USFNA diagnostic studies, and a score of B3 or B4 on the initial USFNA

Diagnostic study	Hemi-thyroidectomy, n (%)	Completion thyroidectomy, n (%)	Total number of operated cases
p-USFNA (B3 or B4)	121 (47.45)	16 (6.27)	255
p-USFNA (B3 or B4) + r-USFNA	36 (37.50)	3 (3.12)	96

*Abbreviations*: *p-USFNA* primary fine-needle aspiration, *r-USFNA* Repeat fine-needle aspiration, *B3* Bethesda score III, *B4* Bethesda score IV

## Discussion

Although previous studies have compared the outcomes of repeat USFNAs with the primary ones, post-operative histopathological data has been missing from all the previous similar studies, except one by VanderLaan [1]. This study is the first to examine the significance of repeating USFNA in patients with p-USFNA diagnosis of B4, and to explore the significance of r-USFNA in guiding the clinical decision of performing hemi-thyroidectomy versus total thyroidectomy for indeterminate nodules. This study aims to address the gap in the literature with regards to correlation of results of repeat USFNA and final surgical pathology. However, there exists a trade off between having definite histopathological data and selecting a well-representative sample from the patient population. Necessitating previous hemi- or total thyroidectomy, as an inclusion criterion in this study, may act to introduce a source of selection bias.

Bethesda categories B3 and B4 comprise a variety of heterogeneous lesions that are neither benign (category B2) nor sufficiently atypical for making a diagnosis of suspicious (category B5) or malignant (category B6) lesions [9–11]. Thus, there is great variability in the literature as to whether patients with such indeterminate diagnoses should undergo surgery or not. Previous investigations have demonstrated that r-USFNA increases the rate of reclassification of indeterminate nodules into one of the more definitive categories of BSRTC and a higher percentage diagnosis of malignancy (categories B5 or B6) [1, 2, 5, 6, 12–15]. However, there exists great variation surrounding the extent of such reclassification in the literature. The percentage of B3 lesions on p-USFNA that upgrade into a more definitive category of B5 or B6 upon performing r-USFNA ranges from 1.35 to 24.77 % with an average of 11.03 % of cases [6, 12, 15–21]. Our data shows that performing a second USFNA results in an upgrade from indeterminate to more definitive categories of Bethesda classification in approximately 20 % of patients, hence changing their clinical management. Specifically, as a result of r-USFNA, surgical intervention was chosen over clinical follow up in 17.3 and 20.0 % of patients with initial BSRTC score of B3 and B4 respectively.

The efficacy of r-USFNA in avoiding a completion thyroidectomy has also been assessed in this study. Diagnostic hemi-thyroidectomy for B3 nodules is not uncommon, and similarly, BSRTC recommends performing lobectomy for B4 lesions. However, our data demonstrates that r-USFNA facilitates choosing the correct surgery (i.e. total thyroidectomy) in about 20 % of nodules that were upgraded from B3/B4 to a more definite malignant category. In these patients, diagnostic hemi- and therapeutic completion thyroidectomies could be avoided. The same concept applies for patients downgraded to a benign diagnosis. In this investigation, 7.4 % of nodules downgraded from B3 to a benign category of B2 after repeating the USFNA. In this group of patients, the clinical management would have changed to a more conservative one not warranting surgery, had they not had a high MTNS score. The proportion of cases in our study that downgraded to a benign score of B2 after r-USFNA are not consistent with those reported in the literature however, ranging from 42.3 to 78.4 % of cases (average 60.3 %) [6, 16–18, 20, 21]. The reason for this discrepancy could be attributed to the very small number of samples in our study that had a repeat USFNA score of B2. In this study, repeating USFNA has been proven to be effective in identifying nodules that are malignant and suspicious for malignancy to undergo total thyroidectomy, but not in ruling-out surgery for benign nodules upon r-USFNA. The reason behind such difference could be due to the fact that all patients in this study had met operative criteria, based on their MTNS score.

In brief, although as per latest guidelines published by the American Thyroid Association, hemi-thyroidectomy may be beneficial in definitive histological diagnosis of indeterminate nodules, repeat USFNA could lead to a clarity of diagnosis preventing the need for lobectomy [22]. In specific, repeat USFNA of an indeterminate nodule may lead to a change to a more conservative management of a benign nodule or upstaging and total thyroidectomy for a malignant lesion. These results have potential implications for cost reduction in the health care system, prevention of patient work absenteeism, and the avoidance of repeat anesthesia.

The analysis of our thyroidectomy database revealed that performing a second USFNA biopsy decreased the number of hemi-thyroidectomy procedures by 9.95 % and prevented a second surgery in approximately 3 % of cases. The discrepancy between the predicted (20 %) and actual (3 %) rate of reduction in completion thyroidectomy surgeries could be attributed to the fact that many of our patients with one USFNA result of B3/B4 underwent total thyroidectomy in the context of a high MTNS score and increased clinical suspicion of malignancy.

Although no data in the literature compared changes in BSRTC categories after repeating USFNA in nodules initially classified as B4, our results for B3 cases on p-USFNA are consistent with the values reported in similar investigations. Specifically, our results show that the proportion of nodules that were reclassified from B3 into B5/B6 after r-USFNA are similar to those reported by VanderLaan (17.8 %), Chen (15.4 %), Yassa (14.16 %) and Baloch (19.42 %) [16, 17, 21, 23].

Our results demonstrate a greater rate of malignancy than what is reported in the BSRTC classification. According to the BSRTC, the risk of malignancy is reported to be 5–15 % for a diagnosis of B3, and 15–30 % for B4 [9]. However, a systematic review has demonstrated a large variation in the risk of malignancy associated with B3 classification, ranging from 6 to 96.7 % (average of 32.7 %) [3, 14, 15, 23]. Likewise, similar issues occur for the B4 classification. In our study, the overall risk of malignancy, as confirmed by the histopathological data, for a thyroid nodule classified as B3 or B4 on p-USFNA was higher than previous reports in the literature, with 58.0 % of B3 and 66.7 % of B4 nodules confirmed to be malignant [1–5, 11]. This difference could be explained by the fact that all the patients included in our study had undergone either hemi- or full thyroidectomy procedure, potentially leading to a selection bias. Furthermore, the heterogeneous nature of B3 and B4 categories and disparities in their interpretations could be considered another factor that has led to the higher rates of malignancies in this study.

The decision to perform surgery in our patient population was not solely limited to the results of the USFNA, but was also influenced by a high MTNS score. MTNS portrays a more accurate account of risk of malignancy by taking into consideration salient factors such as patient demographics, family history and previous radiation exposure. It also incorporates adverse features on ultrasound and on PET scans as an adjunct to the cytological properties of nodules. Thus, an elevated clinical suspicion of malignancy as suggested by the MTNS score, in conjunction with patient and physician preferences, comprise the operative criteria in our institution and guide the clinical decision making.

There are a number of limitations to this study. First, since all of the patients in this investigation have undergone surgery, a selection bias may explain the higher calculated risk of malignancy of B3/B4 nodules. Other limitations include the retrospective nature of the study and the small number of samples that have a repeat USFNA result consistent with a benign nodule (B2).

## Conclusions

Repeating USFNA in patients with indeterminate diagnoses (B3 or B4) increases categorization into more definite diagnostic categories. Close to 20 % of patients are found to have malignant or suspicious thyroid nodules upon repeating the biopsy, hence a diagnostic hemi-thyroidectomy was avoided and a more definitive surgery could be performed. Furthermore, repeat USFNA results in a fewer number of hemi-thyroidectomy and completion thyroidectomy procedures.

## Abbreviations

B2: Bethesda score II
B3: Bethesda score III
B4: Bethesda score IV
B5: Bethesda score V
B6: Bethesda score VI
BSRTC: Bethesda System for Reporting Thyroid Cytopathology
p-USFNA: Primary Fine Needle Aspiration
r-USFNA: Repeat Fine Needle Aspiration
USFNA: Ultrasound Guided Fine Needle Aspiration
